# Diagnosis of *Coxiella burnetii* Cattle Abortion: A One-Year Observational Study

**DOI:** 10.3390/pathogens11040429

**Published:** 2022-04-01

**Authors:** Claude Saegerman, Fabien Grégoire, Laurent Delooz

**Affiliations:** 1 Fundamental and Applied Research for Animal and Health (FARAH) Center, University of Liège, B-4000 Liege, Belgium; laurent.delooz@arsia.be; 2 Regional Association for Animal Registration and Health, B-5530 Ciney, Belgium; fabien.gregoire@arsia.be

**Keywords:** Q fever, *Coxiella burnetii*, cattle, abortion, ELISA, PCR, clinical epidemiology, decision-making

## Abstract

Q fever is a zoonosis occurring worldwide in livestock. Often neglected in differential diagnoses, Q fever can persist in herds causing financial losses. In ruminants, well-known manifestations of Q fever are metritis, infertility, abortion, stillbirth and delivery of a weak or premature calf. In cattle, Q fever is frequently asymptomatic and/or under-reported. Few studies are available on the diagnosis of *Coxiella burnetii* as a cause of abortion in cattle using polymerase chain reaction (PCR) for pathogen detection while enzyme-linked immunosorbent assay (ELISA) is used to assess exposure. Moreover, existing studies include a relatively small number of abortions. The aim of this study is to assess, in the southern part of Belgium, during a year, the performance of diagnosis of *C. burnetii* as a cause of abortion and the putative benefit of enhanced serology using anamnesis (animal patient data, and present, past and environmental history). A one-year random selection of 1212 abortions was analysed both with the PCR method (tissues from fetuses) and two commercialised ELISAs (sera from the mothers). Relative sensitivity and specificity of the ELISA tests were assessed using PCR as the reference test. The prevalence of *C. burnetii* PCR positive was 8.5% (95% CI: 6.99–10.21). The diagnostic value of the ELISA tests was assessed using the area under the receiver operating characteristic curve (AUC-ROC). The sensitivity, specificity and AUC-ROC were similar for both ELISA tests. The diagnostic capacity of the ELISA was confirmed and slightly enhanced if anamnestic information was integrated with a unique scoring index system. A high negative predictive value was demonstrated and a significant reverse association between Ct values and a percentage of the ratio of the optical density between the sample and the positive control (ELISA A or ELISA B) enabling the use of ELISA as an exclusion diagnostic. This study is original by integrating the serological result and the anamnesis in a single index. It opens a new window in enhanced veterinary clinical decision-making.

## 1. Introduction

The causal agent of Q fever is *Coxiella burnetii*, an obligate intracellular Gram-negative bacterium of the Legionellales order [1]. In livestock, the repartition of Q fever is worldwide [2] and domestic ruminants are considered to be the main reservoir for human *C. burnetii* infection [3]. In Belgium, a phylogeography study (years 2009–2019) indicated that the public health risk is mostly found with small ruminants strains [4]. Some limitations of this study were related to the limited data set analysed and the few human samples included. In addition, due to the bulk tank milk monitoring performed only in small ruminants by the Authority after an exceptionally large Q fever outbreak from 2007 to 2010 in the Netherlands, it is logical that some relations can be made mostly between small ruminants (more investigated) and some human cases [5]. However, other studies demonstrated serological evidence of human infection with *C. burnetii* after occupational exposure to aborting cattle (e.g. [6]). Recently, the specific risk that humans acquire *C. burnetii* infections from cattle in an endemically infected area was also strongly suggested [7].

In cattle, Q fever is frequently asymptomatic and/or under-reported. Often neglected in differential diagnoses of reproductive disorders [8], Q fever can persist in herds causing financial losses in the end [1,9]. In ruminants, well-known manifestations of Q fever are metritis, infertility, abortion, stillbirth and delivery of a weak or premature calf [9,10].

A difficulty with *C. burnetii* is that it is frequently endemic in the environment. It will exist in spore form in the dirt for extended periods, in animal reservoirs and in insect reservoirs such as ticks [4]. Early detection of an outbreak should be by PCR to determine the presence and the circulation of *C. Burnetii* and/or by serology (not just looking at IgG, but IgA and IgM and Phase I and II) that will be more related to current or past exposure to *C. bunetii* [9]. Indeed, to control *C. burnetii* shedding in infected cattle herds, several options are effective, such as the use of phase I vaccine [11] and the use of biosecurity and optimised hygiene [12,13]. In Belgium, vaccination of cattle against Q fever is infrequent.

According to the following search string (i.e., keywords and Boolean operator, accessed on December 28, 2021) in PubMed (US National Library of Medicine, National Institutes of Health) “((Q fever) AND (abortion) AND (cattle) AND (ELISA) AND (PCR))”, few studies (*n* = 17) were available on the diagnosis of *C. burnetii* as a cause of abortion in cattle using both polymerase chain reaction (PCR, in relation to the circulation of the bacterium in the herd) and enzyme-linked immunosorbent assay (ELISA, in relation to its exposure). Moreover, existing studies included a relatively small number of abortion cases.

The aim of this study is to properly assess, in field conditions and during a year, with a large dataset, (i) the prevalence of *C. burnetii* as a cause of abortion using PCR, (ii) the performance of serology diagnosis of *C. burnetii* related to the PCR and (iii) the possible benefit of enhanced serology using anamnesis (i.e., animal patient data, and present, past and environmental history). All these objectives contribute to the detection of Q fever in cattle herds and permit the implementation of appropriate mitigation measures.

## 2. Results

### 2.1. Representativeness of Samples

#### 2.1.1. Representativeness of Samples for ELISA Performed All over the Study Period (Sera from the Mothers)

**Figure 1:**
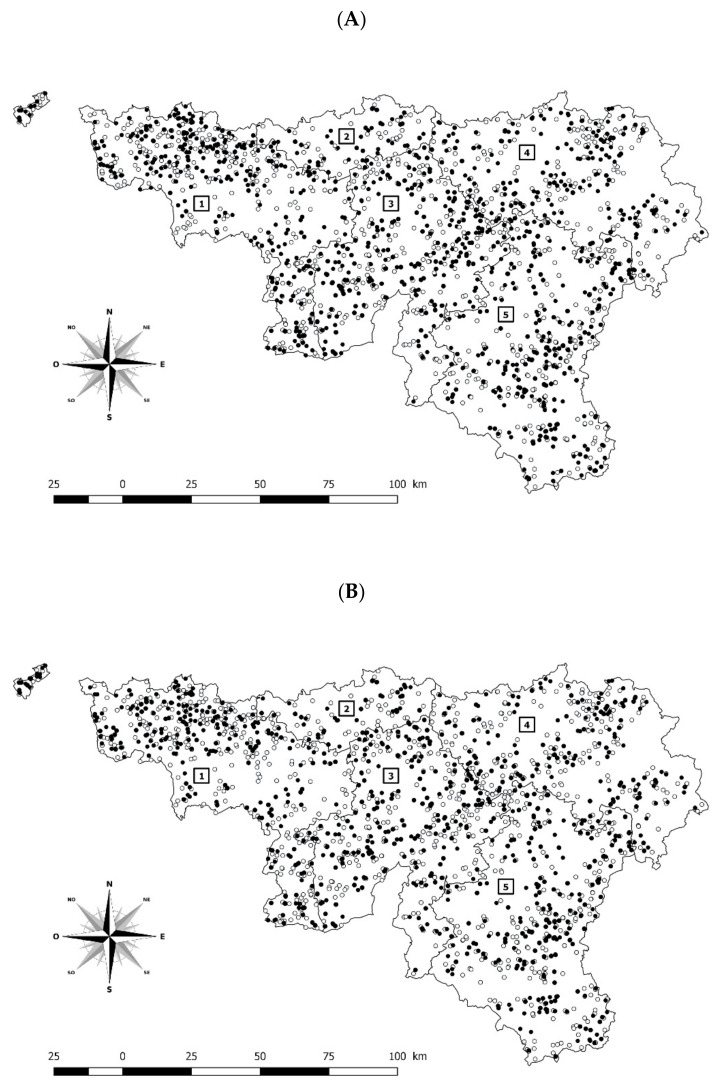
Abortions tested (black dots) or not tested (white dots) by enzyme-linked immunosorbent assay (ELISA—years 2019–2020) (**A**) and by polymerase chain reaction (PCR—the year 2020) in the southern part of Belgium (**B**). Legend: numbers 1 to 5 on the map are related to the provinces; respectively Hainaut, Walloon Brabant, Namur, Liege and Luxembourg. (**A**) Enzyme-linked immunosorbent assay (*n* = 1606). (**B**) Polymerase chain reaction (*n* = 1202).

Based on the sampling frame, no significant difference was observed between samples selected for ELISA (*n* = 1606) and non-selected (*n* = 2070) in the study according to province and week of sampling and taking into account the possible interaction between the two parameters (Logistic regression; *p*-value > 0.10) (Figure 1A). The chi-square of the Hosmer-Lemeshow goodness-of-fit test collapsed on quantiles of estimated probabilities (8 df; α = 0.05) was equal to zero (*p*-value = 1), as a consequence, sample representativeness was considered valid for the ELISA test.

#### 2.1.2. Representativeness of Samples for PCR Performed Only since 2020 (Tissues from Fetuses)

Based on the sampling frame, no significant difference was observed between samples selected for PCR (*n* = 1202) and non-selected (*n* = 462) in the study according to province and week of sampling and taking into account the possible interaction between the two parameters (Logistic regression; *p*-value > 0.16) (Figure 1A). The chi-square of the Hosmer-Lemeshow goodness-of-fit test collapsed on quantiles of estimated probabilities (8 df; α = 0.05) was equal to zero (*p*-value = 1), as a consequence, sample representativeness was considered valid for the PCR test.

### 2.2. Prevalence of Coxiella burnetii Abortion Using Polymerase Chain Reaction

Among 1212 abortions tested, 103 were positive with PCR. The prevalence of abortions due to *C. burnetii* was estimated as 8.50% (95% CI: 6.99–10.21).

### 2.3. Estimation of the Sensitivity, Specificity, Positive and Negative Predictive Values of the Two ELISA Tests Relative to the Polymerase Chain Reaction

In the first scenario, the cut-off recommended by the producers in their notices (for the ELISA A, a ratio of optical density between the sample and the positive control mutiplied by a hundred (% S/P) > 40 and for the ELISA B, a % S/P ≥ 37 was used. In the second scenario, the cut-off used was the one defined using the ROC curve (see next section) (i.e., for the ELISA A, a % S/P > 85 and for the ELISA B, a % S/P ≥ 95).

The estimation of the sensitivity and the specificity, the positive and negative predictive values, and the level of agreement (Kappa coefficient) between PCR and each ELISA test are depicted in Table 1. The parameters studied were in the same order of magnitude for both ELISA A and ELISA B, except for the specificity using the cut-off recommended by the producers. In this case, the specificity was higher for the ELISA A. In addition, in all conditions, the level of agreement between each ELISA test and the PCR was the same and considered negligible (Kappa coefficient less than 0.20). Additionally, the negative predictive value was high whatever the conditions (around 93%).

**Figure 2:**
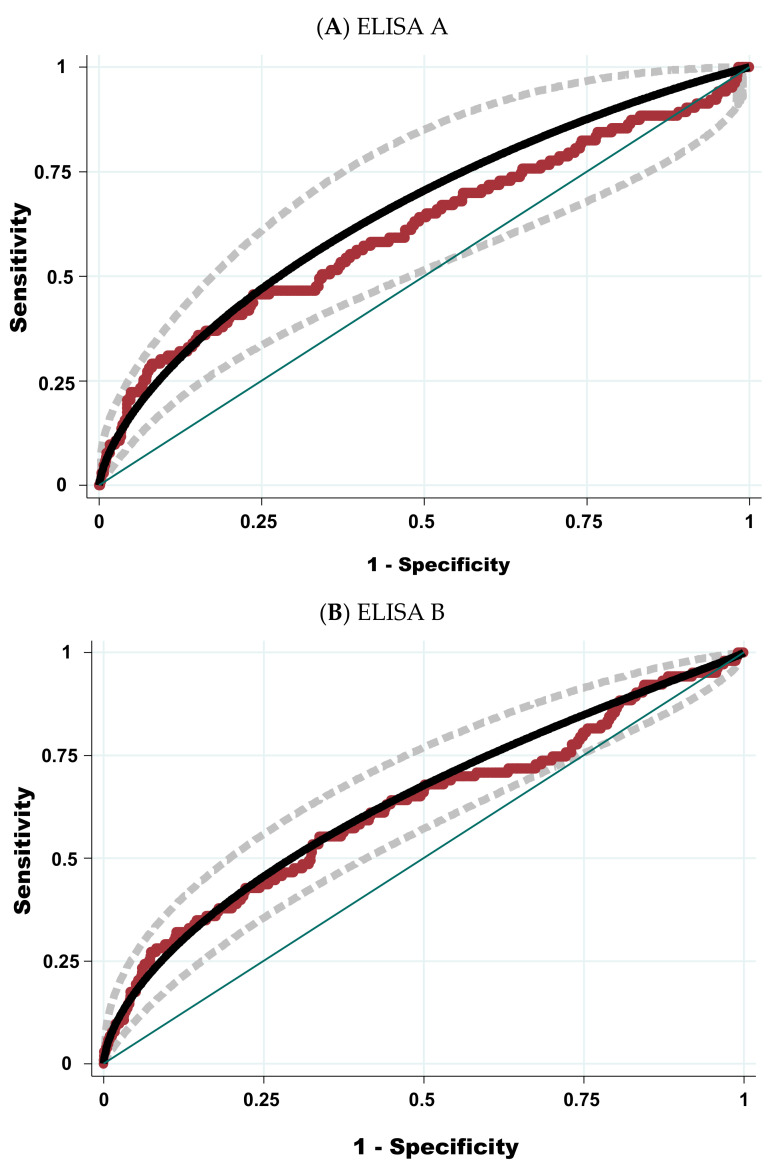
Receiver operating characteristic curve for ELISA A (**A**) and ELISA B (**B**) using the PCR method as reference test. Legend: Dots represent the observed values; the solid curve in black and its 95% confidence interval (broken curves in grey) were fitted according to a binormal distribution. The area under the receiver operating characteristic curve (AUC-ROC) for ELISA A equaled 0.65 (95% CI: 0.56–0.75) with a standard error of 0.05. The goodness-of-fit Chi2 (8 degrees of freedom) for this model = 7.12 (*p*-value = 0.52), indicating that the model is acceptable. The AUC-ROC for ELISA B equal 0.64 (95% CI: 0.58–0.70) with standard error = 0.03. The goodness-of-fit Chi2 (8 degrees of freedom) for this model = 4.49 (*p*-value = 0.81), indicating that the model is good.

**Figure 3:**
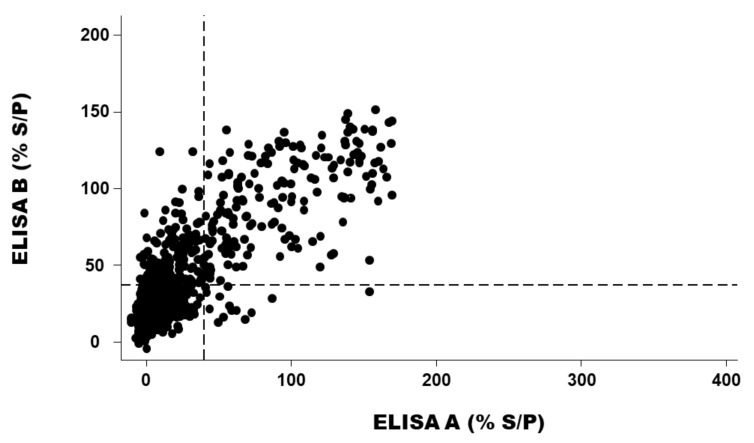
Relation between the results of the ELISA A and the ELISA B expressed as the percentage of the ratio of optical density between the sample and the positive control (% S/P) (*n* = 1606). Legend: Dots represent the observed values; vertical and horizontal broken lines represent the cut-off of ELISA A and ELISA B recommended by the producers, respectively.

In addition, the level of agreement between ELISA A and ELISA B using the cut-off of the producer was good (Kappa coefficient = 0.67 with 95% CI: 0.60–0.67). This level was better using the cut-off determined by the ROC curve (Figure 2): Kappa coefficient = 0.72 with 95% CI between 0.64 and 0.79). The relation between results from ELISA A and ELISA B was also good (Figure 3).

### 2.4. For Positive PCR Results Only, Relation between the Cycle Threshold (Ct) Values of the Sample Result of ELISA A or ELISA B

**Figure 4:**
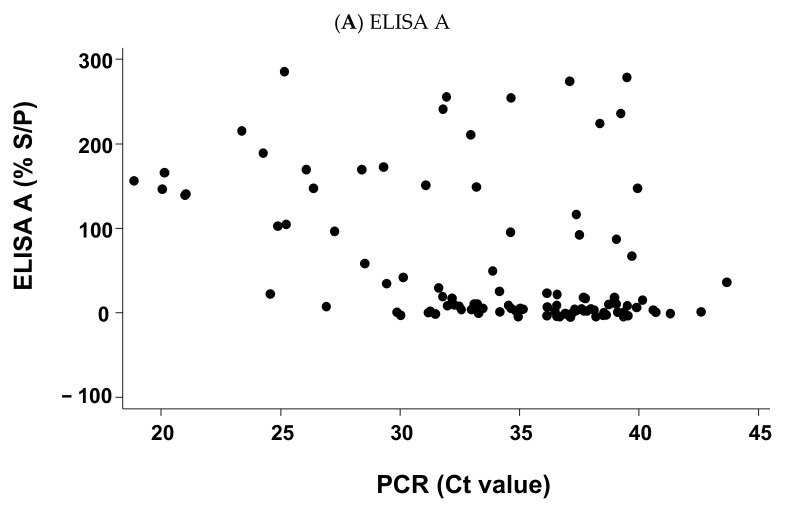

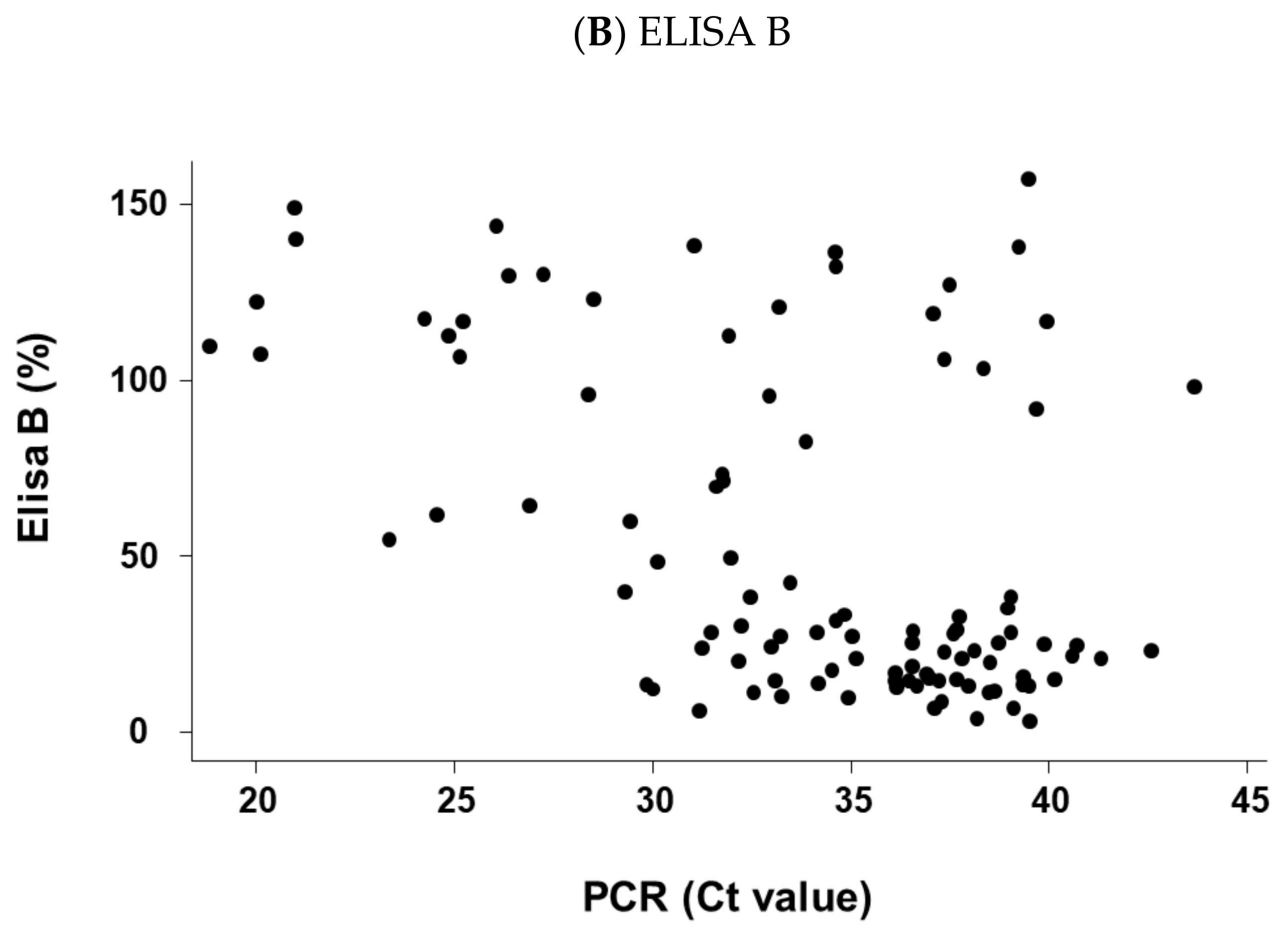
Relation between the cycle threshold (Ct) of the PCR if positive and either result of the ELISA A (**A**) or results of the ELISA B (**B**), expressed as the percentage of the ratio of optical density between the sample and the positive control (% S/P) (*n* = 102). Legend: Dots represent the observed values.

The relation between each ELISA and PCR positive results is depicted in Figure 4. In both cases, inverse correlation was observed but a higher correlation was observed with ELISA B (*n* = 102; Spearman’s rho = −0.406; *p*-value < 0.0001) compared to ELISA A (*n* = 102; Spearman’s rho = −0.388; *p*-value = 0.0001).

### 2.5. Explanatory Variables Related to Q Fever

For 691 samples, the PCR result (including 60 positive, i.e., 8.7%), ELISA result and fully completed anamnesis were available. The PCR result was used as an explicated variable (reference test) and both results of the ELISA tests and of each variable present in the anamnesis as explanatory variables (animal patient data, and present, past and environmental history). Using univariate logistic regression, several explanatory variables were found at risk or protective (Table 2).

### 2.6. Optimisation of the Receiver Operating Characteristic Curve (AUC-ROC) Using Additional Anamnestic Information

#### 2.6.1. Use of Results of ELISA Test Only

The ROC curve for both ELISAs using the PCR as a reference test is depicted in Figure 2. The areas under the ROC curve (AUC) for both ELISA tests were not significantly different (AUC-ROC = 0.65 for ELISA A, with standard deviation (S.D.) = 0.05 versus AUC-ROC = 0.64 for ELISA B, with S.D. = 0.03) but S.D. was smaller for ELISA B.

#### 2.6.2. Mixing Result of ELISA Test and Anamnestic Information to Increase the Area under the Receiver Operating Characteristic Curve

**Figure 5:**
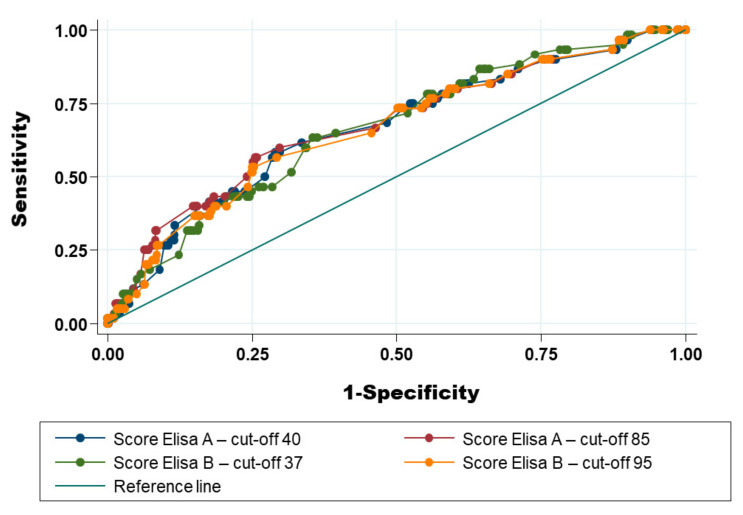
ROC curve for the four different scoring systems depending on the ELISA test and associated cut-off considered and using the PCR method as a reference test. Legend: The area under the receiver operating characteristic curve (AUC-ROC) for ELISA A and cut-off 40 equal 0.66 (95% CI: 0.59–0.74) with standard error = 0.04. The AUC-ROC for ELISA A and cut-off 85 equal 0.68 (95% CI: 0.60–0.75) with standard error = 0.04. The AUC-ROC for ELISA B and cut-off 37% equal 0.66 (95% CI: 0.59–0.73) with standard error = 0.04. The AUC-ROC for ELISA B and cut-off 95% equal 0.66 (95% CI: 0.59–0.74) with standard error = 0.04.

Using the following algorithm, four different additional ROC curves were produced using both ELISAs with the cut-off recommended by the producers and the cut-off determined by the ROC curves in Figure 5.

The algorithm aggregates the results presented in the Table 2 in order to generate a unique score:

Score = (Result ELISAi × OR ELISAi) + (si(C5 = “Natural mating”; 1/0.45; 0)) + (si(C6 = “ Free-stall on slatted floors”; 1*3.45; si(C6 = “ Free-stall on straw”; 1 × 2.55; 0))) + (C7 × 2) + (C8 × 4.85) + (C9 × 2.22) + (C10 × 1.83)(1)

With: ELISAi, ELISA is defined in Table 2 with the following codification, C1 or C2 or C3 or C4 (i.e., four different scores were calculated); OR, odds ratio; C5 to C10, refer to the codification of each risk or protective factor (see column “code” in Table 2 for the details); ×, multiplication.

The inclusion of anamnestic information allows a slight increase of the AUC-ROC, whichever ELISA or cut-off was used. The AUC-ROC of the four different scoring systems was not significantly different (Chi2 (3 d.f.; α = 0.05) = 3.33 with a *p*-value of 0.34). The best cut-off for all of the AUC-ROC was a score = 7.5.

## 3. Discussion

A total of 3676 cases of abortion were submitted to the laboratory in a year. Following the Belgian statistical office (https://statbel.fgov.be/; accessed on 28 December 2021), 52.32% of the bovine population are females of 2 years old or more (*n* = 581,603), from which 72.32% are in production (*n* = 422,946). Knowing that around 1.9% of the pregnant bovine females abort [14], the total number of abortions can be estimated at 8036 per year. Indeed, a rough estimation of the overall surveillance sensitivity of abortions was 46% (i.e., 3676/8036). This overall surveillance sensitivity is higher than previously reported by [15] in the literature (i.e., 34% in France). This benchmarking indicates relatively good surveillance of abortions in the southern part of Belgium. The ratio of the reported abortion at the herd level and the expected number of abortions using the above reasoning should be proposed as an epidemiological indicator of the quality of the overall surveillance.

Using numerous samples of bovine abortions (*n* = 1202) from the passive surveillance in southern Belgium, this study allows estimating the percentage of abortions due to *C. burnetii* as 8.5% with a short 95% CI (6.99–10.21) on the basis of the PCR test. Moreover, the representativeness of the sampling was validated in space and time. This percentage is compatible with the percentage observed previously in Italy (11.6% with 95% CI: 6.77–18.14) and in Brazil (9.21% with 95% CI: 3.78–18.06) [16,17]. Due to the fact that the presence of *C. burnetii* is endemic worldwide with the exception of New Zealand [18,19], the contribution of *C. burnetii* in bovine abortions calculated in this study is a robust point estimate.

Despite the lack of a gold standard test for Q fever, its diagnosis in cattle is mainly made by PCR and ELISA, enabling the differentiation of the different causes of abortion [20,21]. In this study, the PCR was considered the first-line reference test for the diagnosis of abortions for three reasons. First, the transmission of the samples within 48 h after abortion prevents a maximum of false-negative results [22]. Second, a positive PCR test on a foetal tissue sample refers to a recent presence/circulation of *C. burnetii* in the herd permitting the adoption of mitigation measures. It must be noted that a positive ELISA result on a dam that has aborted corresponds only to a past recent or past exposure to the *C. burnetii* [9]. Third, the isolation of the bacteria is difficult for *C. burnetii* and less sensitive than PCR, as demonstrated in humans with spontaneous abortions [23]. With this prerequisite, it will be very interesting to estimate the sensitivity, specificity, positive and negative predictive values and level of agreement between each ELISA test and the PCR as a reference test, for the specific purpose of diagnosis of cattle abortion. The main results indicate a relatively good specificity of the ELISA tests with regard to the PCR (between 86 and 93%), whatever the cut-off used (from the producer or determined using the ROC curve). In addition, the sensitivity was relatively low, between 33% (ELISA A) and 43% (ELISA B) using the cut-off recommended by the producer. A previous study based on both phases I and II-ELISA confirmed this low sensitivity [24].

Another important (but it depends on the true prevalence of *C. burnetii* in aborted dams) fact is that the negative predictive value (NPV) was high in all conditions. This is interesting because the NPV is the confidence attributed to a negative result (around 93%). Indeed, in cases of ELISA negative results, an exclusion diagnostic of abortion due to *C. burnetii* can be postulated (with little risk of error). However, other causes of abortion should be screened. Moreover, the level of agreement between the results of the two ELISA tests is good, as is the relation between the % S/P from ELISA A and ELISA B.

In addition, for positive samples in PCR (with Ct value available), a significant reverse relation was observed between the Ct values in PCR and the % S/P for ELISA A and for ELISA B. This relation was higher for ELISA B.

Indeed, in developed countries and based on the three previous paragraphs, we recommend in the case of cattle abortion, the preferential use of PCR on tissues from fetuses. It is justified by the fact that positive PCR is related to the presence or circulation of *C. burnetii* in the herd. In the case of developing countries and if PCR is too expensive or not available, we recommend the use of ELISA phases I and II to exclude the Q fever due to the high specificity and associated high negative predictive value of this test. However, a positive ELISA is only related to the present or past exposure of cattle to *C. burnetii*. In addition, more research is needed to investigate the possible benefit to use a specific phase ELISA for the purpose of the detection of cattle abortion.

Exploratory variables that predict a positive PCR result (i.e., presence or circulation of *C. burnetii* in the herd) were identified. Mitigation measures can be used to minimize (risk factors) or to optimize (protective factors) those predictors. There are respectively 3.03 and 4.65 times more chance of having a positive PCR test if the ELISA A test is positive considering the cut-off determined by the kit producer or determined by the ROC curve. Additionally, there are respectively 2.52 and 4.68 times more chance of having a positive PCR test if the ELISA B test is positive considering the cut-off determined by the kit producer or determined by the ROC curve. Using logistic regression analysis, we also investigated all variables included in the anamnesis done by the veterinarians and found one protective and six risk factors. The protective factor is natural in mating. This was opposite to a previous finding performed in Algeria [25], in which artificial insemination was found as a protective factor. However, it is not univocal in the literature since artificial insemination by people other than artificial insemination technicians is high risk (OR = 7.7) [26]. More investigations are needed to clarify the contribution of this variable.

Risk factors identified are free-stall on slatted floors, free-stall on straw, pasture surrounded by trees or hedges, contact with goats, a proportion of dairy in the herd, and the use of well water in winter. It must be noted that the highest odds ratio was found for contact with goats.

Free-stall (without other precision) was previously cited as a protective factor in a study but it was not focused on abortion [9]. However, the interaction between the housing system and herd size was also strongly evidenced in another study [27] and herd size was frequently cited as a risk factor for Q fever in cattle (e.g., [12,28]. Further investigations should be devoted to elucidating this explanatory variable.

Pastures surrounded by trees or hedges should be related to the presence of ticks (e.g., [29]) and ticks may play a role in the transmission of *C. burnetii* infection [30]. Tick presence in cows was also cited as a risk factor for Q fever in dairy herds [12]. In addition, in hyperendemic Q fever areas, living in a house nearer to the forest was considered a risk factor for human Q fever [31]. Contact with goats was frequently cited as a source or suggested as a risk factor for *C. burnetii* in humans (e.g., [4,30]) or in cattle [32,33]. A higher proportion of dairy in a herd at risk should be related to the fact that a previous study has found Holstein breed at risk for Q fever [34]. Drinking well water was also previously identified as a risk factor [9]. Well water is sometimes used on a farm as a source of water because it is less costly. Well water could be contaminated by *C. burnetii* by animal shedders in the herd or infected rodents [9]. In addition, *C. burnetii* is resistant in the environment for a long time and its infectious dose is low [35].

Combining both the result of the ELISA tests and significant protective and risk variables of the anamnestic questionnaire using a unique scoring system allows a slight increase of the AUC-ROC. This proposed decision-making method mimics and supports the reasoning of veterinarians faced with the interpretation of the laboratory diagnostic results at the farm level. The awareness of the veterinarians of the utility of completing those anamneses is a key condition in order to enhance the power of diagnostic tools in integrating both laboratory results and anamnestic information related to specific herd conditions for better decision-making. Further prospects in terms of artificial intelligence are close to the door (e.g., the development of an application directly available in real-time by the vets). Accurate diagnosis permits the implementation of appropriate control measures to avoid the persistence of Q fever in the herd limit its spread (e.g., vaccination, biosecurity measures), and limit exposure to humans by using personal protective equipment.

In addition, with exception (e.g., [25]), most of the studies on risk and protective factors of Q fever in cattle are based on cross-sectional serological surveys (related to recent or past exposure to *C. burnetii*) and not specifically on clinical signs such as abortion (more related to the presence or circulation of *C. burnetii*) which are riskier.

Some limitations are inherent in this field study, as no gold standard test exists for the diagnosis of Q fever [20,21]. The anamnestic questionnaire used in the case of abortion is not intended only for the study of Q fever but, on the contrary, is to generally capture important findings related to the most important causes of cattle abortion [36]. Updating the main risk and protective factors for most important abortive pathogens in cattle is recommended with a focus on systematic reviews and meta-analyses. This should improve the success in targeting important risk or protective factors pertinent to pathogens in the study area. Another limitation is related to the non-completion of around half of the anamneses submitted by veterinarians.

## 4. Materials and Methods

### 4.1. Study Area

This study was conducted in the southern part of Belgium, including five different provinces (Walloon Brabant, Hainaut, Namur, Liege and Luxembourg). The southern part of Belgium covers 16,901 km^2^ (55.1% of Belgium). In the study area, 9608 cattle herds with 1,111,716 bovines were registered in 2020 [37].

### 4.2. Sampling Design

**Figure 6:**
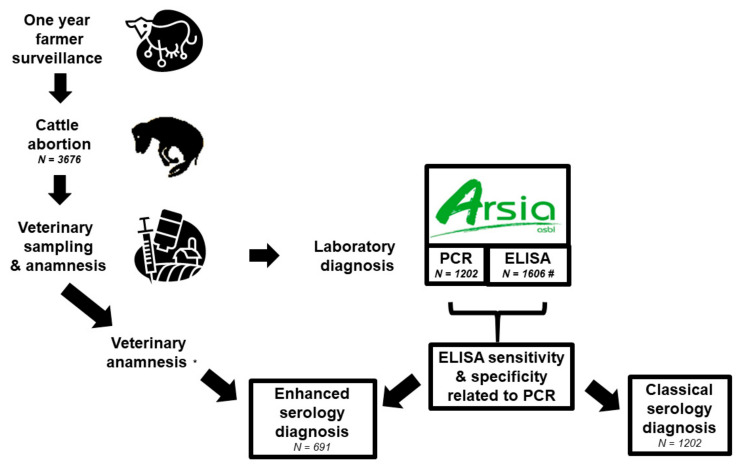
Sampling design of the study. Legend: ELISA, enzyme-linked immunosorbent assay; PCR, polymerase chain reaction; * Anamnesis (animal patient data, and present, past and environmental history) was completed for 800 samples submitted to serology (i.e., 49.81% with 95% CI: 47.34–52.29) for which 691 samples were both tested by ELISA and PCR; # For 1202 sera, both results of ELISA and PCR are available.

Samples (i.e., tissues from the fetuses and the sera from the mothers) were obtained in the context of the passive surveillance programme for abortion and collected by the local veterinary practitioner within 48 h after abortion (Royal Decree of 28/02/1999, on special measures for the epidemiological surveillance and prevention of notifiable cattle diseases) (Figure 6). This time period prevents false-negative results in the polymerase chain reaction method [22].

Samples were sent together with a standardized anamnesis form completed by the veterinarian (https://www.arsia.be/wp-content/uploads/documents-telechargeables/Form45.pdf; accessed on 28 December 2021) to the regional laboratory (Regional Association of Animal Health and Identification, ARSIA).

Only the bovine abortion cases (tissues of fetuses) submitted to the laboratory from 31 July 2019 to 12 August 2020 together with at least the maternal serum and the aborted foetus were included in this study. A total of 3676 cases of abortion were submitted to the laboratory of which 1606 were randomly selected for future analyses by enzyme-linked immunosorbent assay (ELISA) (Figure 1A). Polymerase chain reaction (PCR) was only performed from the beginning of 2020 (*n* = 1212) (Figure 1B).

Mothers were on average 50 months old (standard deviation: 20 months; min-max: 15–171 months) and their speculation is respectively meat for 70% of the cases, milk for 25% and mixt for the remaining 5%.

STARD (for studies of diagnostic accuracy) and STROBE (for observational studies in epidemiology) guidelines of the equator network were followed (Supplementary Files S1 and S2).

### 4.3. Detection of Coxiella burnetii Using Polymerase Chain Reaction (PCR)

For deoxyribonucleic acid (DNA) extraction, approximately 20 mg of roughly foetal tissues (pool of spleen and, if available, placental cotyledons) were mechanically disrupted using a Mixer Mill 400 grinder (Restch^®^, Germany). Lysis buffer and proteinase K (Indical Bioscience, Germany) were added to the samples, and lysis was performed by incubating at 70 °C +/−5 °C for 30 min. Lysates were then homogenised and centrifuged for 1 min at 6000× *g*. The DNA purification was performed using an automat KingFisher Flex 96TM with the dedicated buffers contained in the IndiMag^®^ Pathogen Kit (Indical Bioscience, Germany).

*C. burnetii*-specific DNA in the samples was detected by amplification IS1111 elements with a real-time polymerase chain reaction (LSI VetMAX™ *C. burnetii*, Thermo Fisher Scientific, Germany). Cycle threshold (Ct) values ≤ 40 were considered as positive and Ct values > 40 as negative.

The method was validated through yearly proficiency tests organised by the Belgian National Reference Laboratory (Sciensano) since 2017. The use of negative and positive controls, including a positive field sample whose results are followed on a quality control card, enables the validation of the PCR runs.

### 4.4. Enzyme-Linked Immunosorbent Assay (ELISA) Tests

#### 4.4.1. First Indirect ELISA Test [A]

Each blood sample was tested using the PrioCHECK™ Ruminant Q Fever Ab Plate Kit (Thermo Fisher Scientific, Waltham, MA, USA). The ELISA antigen was recovered from a C. *burnetii* strain isolated from an aborted sheep in France. A cocktail of both antigen phases (I and II) were used in this assay to detect total immunoglobulins G (Ig G) anti-*C. burnetii*. Briefly, the serum samples were diluted 1/400 for testing as indicated by the manufacturer. The negative and positive controls were always included when serum samples were examined. Five microliters of sample were necessary. After a 60-min incubation period at 37.8 °C, the plates were washed with phosphate buffer saline before the addition of horseradish peroxidase (HRP) conjugated antibody. The results were expressed in optical density sample/positive control (S/P) ratio:

S/P = (OD sample − OD average NC)/(OD average PC − OD average NC)(2)

With: OD, optical density; NC, negative control; PC, positive control.

The testing procedure is valid if the following conditions are respected:

OD average PC > 0.4(3)

And

OD average PC/OD average NC > 2(4)

% S/P = S/P × 100(5)

As recommended by the producer, a serum sample was considered positive when % S/P > 40.

#### 4.4.2. Second Indirect ELISA Test [B]

Each blood sample was tested using the Monoscreen Ab ELISA *C. burnetii* (BIO K 298/2, Bio-X Diagnostics, Rochefort, Belgium). The ELISA antigen was recovered from a *C. burnetii*, referent Nine Mile strain. A cocktail of both antigen phases (I and II) were used in this assay to detect total immunoglobulins G (Ig G) anti-*C. burnetii*. Briefly, the serum samples were diluted 1/100 for testing as indicated by the manufacturer. The negative and positive controls were always included when serum samples were examined. Twenty microliters of sample were necessary. After a 60-min incubation period at room temperature (21 °C +/−3 °C), the plate was washed with washing solution before the addition of protein G conjugated antibody coupled to the Raifort peroxidase. After a second incubation at room temperature (21 °C +/−3 °C) for one more hour, the plate is revealed by the addition of 3,3′,5,5′-tetramethylbenzidine (TMB) for 10 min at room temperature. The reaction is stopped by the addition of a blocking solution. The results were expressed in optical density S/P ratio (Equation (2)) and % S/P (Equation (5)):

The testing procedure is valid if the two following conditions are respected:

(OD average PC − OD average NC) > 1 (6)

And

OD average NC < 0.4 (7)

As recommended by the producer, a serum sample was considered positive when % S/P ≥ 37.

### 4.5. Statistical Analysis

#### 4.5.1. Basis Statistics

Representativeness has been defined as the degree of similarity of a study population compared to an external population. In this survey, the representativeness of the abortion cases (both fetuses and mother’s sera) included in the study was indirectly verified with respect to the location (province) and the sampling date and using a logistic regression model including the interaction of variables. The fitting of the model was assessed using the chi-square of the Hosmer-Lemeshow goodness-of-fit test collapsed on quantiles of estimated probabilities [38].

The confidence interval (95% CI) of prevalence was estimated using an exact binomial distribution.

The calculation of the odds ratio was used to check the relation between the PCR result on the aborted foetus and the explanatory variables of the anamnesis and the results of the two ELISA [39]. A significant risk or protective variables were retained to construct a further scoring index system (see the section of Results for the equation of the score).

The level of agreement between tests was assessed using the Kappa coefficient [39].

For positive PCR samples, the relation between the Ct value and the % S/P (ELISA A and ELISA B) was assessed using Spearman’s rho coefficient [39].

Statistical analyses were performed using STATA/SE Acad. 14.2 (Stata Corp., College Station, TX, USA).

#### 4.5.2. Receiver Operating Characteristic Curve

A ROC curve (probability curve) was plotted with true-positive results (Y-Axis) against the false-positive results (X-Axis). The AUC-ROC is the performance measurement for the classification of the ELISA results at various thresholds settings. The higher the AUC-ROC, the better the ELISA test is able to distinguish between confirmed and unconfirmed C. *burnetii* PCR results (i.e., measurement of the separation of the two sub-populations). In addition, Youden’s index “J” is frequently used in conjunction with the ROC curve analysis to estimate the best cut-off [39], with:

Youden’s index = sensitivity + specificity − 1 (8)

The value of AUC-ROC ranges from 0 to 1 (inclusive). A zero value is observed when a diagnostic test gives the same proportion of positive results for groups of confirmed or unconfirmed *C. burnetii* PCR results. A value of 1 indicates that there are no false positives or false negatives, i.e., that the test is perfect.

## 5. Conclusions

The prevalence of cattle abortion due to *C. burnetii* in the southern part of Belgium was estimated at 8.5% with the accuracy and representativeness of samples valid. The relative performance of ELISA tests was assessed for the purpose of an abortion diagnostic using the area under the receiver operating characteristic curve and the PCR as a reference test. Considering their relative sensitivity and specificity, a high negative predictive value was demonstrated and, for positive PCR results, a significant reverse association between Ct values and either % S/P (ELISA A and ELISA B) enabling the use of ELISA test as exclusion diagnostic. Integrating both the serological result and anamnesis in a single score is original and opens a new window for enhanced veterinary clinical decision-making.

## Figures and Tables

**Table 1:** Sensitivity, specificity, positive and negative predictive values of ELISA A and ELISA B depending on the cut-off used and with the polymerase chain reaction as a reference test.

Scenario and Cut-Off Used	Sensitivity in % (95% CI)	Specificity in % (95% CI)	Positive Predictive Value in % (95% CI)	Negative Predictive Value in % (95% CI)	Coefficient Kappa (95% CI)
ELISA [A] Cut-off: % S/P > 40	33.01 (24.06–42.97)	86.02 (83.84–88.01)	17.99 (12.79–24.22)	93.26 (91.54–94.71)	0.14 (0.08–0.19)
ELISA [A] Cut-off: % S/P > 85	28.16 (19.73–37.87)	91.88 (90.12–93.42)	24.37 (16.97–33.09)	93.23 (91.57–94.65)	0.19 (0.13–0.24)
ELISA [B] Cut-off: % S/P > 37	42.72 (33.02–52.85)	77.19 (74.60–79.63)	14.81 (10.98–19.37)	93.55 (91.18–95.06)	0.11 (0.06–0.15)
ELISA [B] Cut-off: % S/P > 95	27.18 (18.88–36.84)	92.60 (90.90–94.08)	24.45 (17.63–34.65)	93.19 (91.54–94.61)	0.11 (0.06–0.15)

Legend: % S/P is the ratio between the optical density between the sample and the positive control expressed as a percentage.

**Table 2:** Significant explanatory variables at risk or protective using univariate binary logistic regression.

Variable	Code	Modality	OR	(95% CI)	*p*-Value	At Risk or Protective
ELISA [A] using the cut-off of the producer (% S/P > 40)	C1	Negative	Ref	-	-	
		Positive	3.03	(1.94–4.73)	<0.001	At risk
ELISA [A] using the cut-off determined by the ROC curve (% S/P > 85)	C2	Negative	Ref	-	-	
		Positive	4.65	(2.89–7.49)	<0.001	At risk
ELISA [B] using the cut-off of the producer (% S/P > 37)	C3	Negative	Ref	-	-	
		Positive	2.52	(1.67–3.82)	<0.001	At risk
ELISA [B] using the cut-off determined by the ROC curve (% S/P > 95)	C4	Negative	Ref	-	-	
		Positive	4.68	(2.87–7.62)	<0.001	At risk
AI or natural mating	C5	1 or 2 AI	Ref	-	-	
		>2 AI	1.32	(0.45–3.88)	0.75	
		Natural mating	0.45	(0.26–0.78)	0.005	Protective
Winter stabling of animals	C6	Tethered housing	Ref	-	-	
		Free-stall on slatted floors (A)	3.45	(1.31–9.11)	0.012	At risk
		Free-stall on straw (B)	2.55	(1.09–5.95)	0.03	At risk
		(A) and (B)	7.97	(0.30–215.21)	0.22	
Pasture surrounded by trees or hedges	C7	No	Ref	-	-	
		Yes	2.00	(1.02–3.93)	0.044	At risk
Contact with goats	C8	No	Ref	-	-	
		Yes	4.85	(1.22–19.26)	0.025	At risk
Proportion of dairy in the herd	C9	<20%	Ref	-	-	
		≥20%	2.22	(1.30–3.78)	0.004	Risk
Use of well water in winter	C10	No	Ref	-	-	
		Yes	1.83	(1.02–3.28)	0.041	Risk

Legend: CI, confidence interval; Ref, reference; AI, artificial insemination.

## Data Availability

The data that support the findings of this study are available from the corresponding author upon reasonable request.

## Supplementary Materials

The following supporting information can be downloaded at: https://www.mdpi.com/article/10.3390/pathogens11040429/s1, File S1: STARD Checklist (for Studies of Diagnostic Accuracy); File S2: STROBE Checklist (for Observational Studies in Epidemiology).
