# Supplementary material for: Diagnosis of Coxiella burnetii Cattle Abortion: A One-Year Observational Study
Source: Pathogens. 2022 Apr 1;11(4):429. doi: 10.3390/pathogens11040429 (PMC9032501; doi:10.3390/pathogens11040429)
Supplement: Supplementary file 1 [file pathogens-11-00429-s001.zip › pathogens-1587355-supplementary.pdf]

Supplementary File S1. STARD CHECKLIST (FOR STUDIES OF DIAGNOSTIC ACCURACY)

Section & Topic	No	Item	Reported on page #
**TITLE OR ABSTRACT**		**Diagnosis of Coxiella burnetii cattle abortion: a one-year observational study**	
	**1**	Identification as a study of diagnostic accuracy using at least one measure of accuracy (such as sensitivity, specificity, predictive values, or AUC)	1
**ABSTRACT**			
	**2**	Structured summary of study design, methods, results, and conclusions (for specific guidance, see STARD for Abstracts)	1
**INTRODUCTION**			
	**3**	Scientific and clinical background, including the intended use and clinical role of the index test	2
	**4**	Study objectives and hypotheses	2
**METHODS**			
Study design	**5**	Whether data collection was planned before the index test and reference standard were performed (prospective study) or after (retrospective study)	12-13
Participants	**6**	Eligibility criteria	12-13
	**7**	On what basis potentially eligible participants were identified (such as symptoms, results from previous tests, inclusion in registry)	12-13
	**8**	Where and when potentially eligible participants were identified (setting, location and dates)	12-13
	**9**	Whether participants formed a consecutive, random or convenience series	12-13
Test methods	**10a**	Index test, in sufficient detail to allow replication	14
	**10b**	Reference standard, in sufficient detail to allow replication	14
	**11**	Rationale for choosing the reference standard (if alternatives exist)	14
	**12a**	Definition of and rationale for test positivity cut-offs or result categories of the index test, distinguishing pre-specified from exploratory	14
	**12b**	Definition of and rationale for test positivity cut-offs or result categories of the reference standard, distinguishing pre-specified from exploratory	14
	**13a**	Whether clinical information and reference standard results were available to the performers/readers of the index test	14
	**13b**	Whether clinical information and index test results were available to the assessors of the reference standard	14
Analysis	**14**	Methods for estimating or comparing measures of diagnostic accuracy	14
	**15**	How indeterminate index test or reference standard results were handled	14
	**16**	How missing data on the index test and reference standard were handled	14
	**17**	Any analyses of variability in diagnostic accuracy, distinguishing pre-specified from exploratory	14
	**18**	Intended sample size and how it was determined	14
**RESULTS**			
Participants	**19**	Flow of participants, using a diagram	12 (figure 6)
	**20**	Baseline demographic and clinical characteristics of participants	3-4 and 13
	**21a**	Distribution of severity of disease in those with the target condition	12
	**21b**	Distribution of alternative diagnoses in those without the target condition	8-9
	**22**	Time interval and any clinical interventions between index test and reference standard	12
Test results	**23**	Cross tabulation of the index test results (or their distribution) by the results of the reference standard	4
	**24**	Estimates of diagnostic accuracy and their precision (such as 95% confidence intervals)	4
	**25**	Any adverse events from performing the index test or the reference standard	-
**DISCUSSION**			
	**26**	Study limitations, including sources of potential bias, statistical uncertainty, and generalisability	11-12
	**27**	Implications for practice, including the intended use and clinical role of the index test	9-12
**OTHER INFORMATION**			

CC BY SA

	**28**	Registration number and name of registry	12
	**29**	Where the full study protocol can be accessed	12 (figure 6)
	**30**	Sources of funding and other support; role of funders	15

Supplementary File S2. STROBE STATEMENT—CHECKLIST (FOR OBSERVATIONAL STUDIES)

	**Item No**	**Recommendation**	**Page No**
**Title and abstract**	1	(*a*) Indicate the study's design with a commonly used term in the title or the abstract	1
		(*b*) Provide in the abstract an informative and balanced summary of what was done and what was found	1
**Introduction**			
Background/rationale	2	Explain the scientific background and rationale for the investigation being reported	2
Objectives	3	State specific objectives, including any prespecified hypotheses	2
**Methods**			
Study design	4	Present key elements of study design early in the paper	12
Setting	5	Describe the setting, locations, and relevant dates, including periods of recruitment, exposure, follow-up, and data collection	12
Participants	6	(*a*) Give the eligibility criteria, and the sources and methods of selection of participants	12
Variables	7	Clearly define all outcomes, exposures, predictors, potential confounders, and effect modifiers. Give diagnostic criteria, if applicable	12-13
Data sources/ measurement	8*	For each variable of interest, give sources of data and details of methods of assessment (measurement). Describe comparability of assessment methods if there is more than one group	12
Bias	9	Describe any efforts to address potential sources of bias	14
Study size	10	Explain how the study size was arrived at	12

Quantitative variables	11	Explain how quantitative variables were handled in the analyses. If applicable, describe which groupings were chosen and why	13-14
Statistical methods	12	(*a*) Describe all statistical methods, including those used to control for confounding	14-15
		(*b*) Describe any methods used to examine subgroups and interactions	14
		(*c*) Explain how missing data were addressed	14
		(*d*) If applicable, describe analytical methods taking account of sampling strategy	14
		(*e*) Describe any sensitivity analyses	14
**Results**			
Participants	13*	(a) Report numbers of individuals at each stage of study—eg numbers potentially eligible, examined for eligibility, confirmed eligible, included in the study, completing follow-up, and analysed	2-4
		(b) Give reasons for non-participation at each stage	2-4
		(c) Consider use of a flow diagram	Already present at page 12
Descriptive data	14*	(a) Give characteristics of study participants (eg demographic, clinical, social) and information on exposures and potential confounders	2-4
		(b) Indicate number of participants with missing data for each variable of interest	Already present in page 12
Outcome data	15*	Report numbers of outcome events or summary measures	3-10
Main results	16	(*a*) Give unadjusted estimates and, if applicable, confounder-adjusted estimates and their precision (eg, 95% confidence interval). Make clear which confounders were adjusted for and why they were included	3-10
		(*b*) Report category boundaries when continuous variables were categorized	7
		(*c*) If relevant, consider translating estimates of relative risk into absolute risk for a meaningful time period	Not relevant
Other analyses	17	Report other analyses done—eg analyses of subgroups and interactions, and sensitivity analyses	2-3

**Discussion**			
Key results	18	Summarise key results with reference to study objectives	9-11
Limitations	19	Discuss limitations of the study, taking into account sources of potential bias or imprecision. Discuss both direction and magnitude of any potential bias	11
Interpretation	20	Give a cautious overall interpretation of results considering objectives, limitations, multiplicity of analyses, results from similar studies, and other relevant evidence	9-11
Generalisability	21	Discuss the generalisability (external validity) of the study results	9-11
**Other information**			
Funding	22	Give the source of funding and the role of the funders for the present study and, if applicable, for the original study on which the present article is based	15

*Give information separately for exposed and unexposed groups.

Note: An Explanation and Elaboration article discusses each checklist item and gives methodological background and published examples of transparent reporting. The STROBE checklist is best used in conjunction with this article (freely available on the Web sites of PLoS Medicine at http://www.plosmedicine.org/, Annals of Internal Medicine at http://www.annals.org/, and Epidemiology at http://www.epidem.com/). Information on the STROBE Initiative is available at www.strobe-statement.org.

CC BY SA

STARD 2015

AIM

STARD stands for “Standards for Reporting Diagnostic accuracy studies”. This list of items was developed to contribute to the completeness and transparency of reporting of diagnostic accuracy studies. Authors can use the list to write informative study reports. Editors and peer-reviewers can use it to evaluate whether the information has been included in manuscripts submitted for publication.

EXPLANATION

A **diagnostic accuracy study** evaluates the ability of one or more medical tests to correctly classify study participants as having a **target condition.** This can be a disease, a disease stage, response or benefit from therapy, or an event or condition in the future. A medical test can be an imaging procedure, a laboratory test, elements from history and physical examination, a combination of these, or any other method for collecting information about the current health status of a patient.

The test whose accuracy is evaluated is called **index test.** A study can evaluate the accuracy of one or more index tests. Evaluating the ability of a medical test to correctly classify patients is typically done by comparing the distribution of the index test results with those of the **reference standard**. The reference standard is the best available method for establishing the presence or absence of the target condition. An accuracy study can rely on one or more reference standards.

If test results are categorized as either positive or negative, the cross tabulation of the index test results against those of the reference standard can be used to estimate the **sensitivity** of the index test (the proportion of participants *with* the target condition who have a positive index test), and its **specificity** (the proportion *without* the target condition who have a negative index test). From this cross tabulation (sometimes referred to as the contingency or “2x2” table), several other accuracy statistics can be estimated, such as the positive and negative **predictive values** of the test. Confidence intervals around estimates of accuracy can then be calculated to quantify the statistical **precision** of the measurements.

If the index test results can take more than two values, categorization of test results as positive or negative requires a **test positivity cut-off**. When multiple such cut-offs can be defined, authors can report a receiver operating characteristic (ROC) curve which graphically represents the combination of sensitivity and specificity for each possible test positivity cut-off. The **area under the ROC curve** informs in a single numerical value about the overall diagnostic accuracy of the index test.

The **intended use** of a medical test can be diagnosis, screening, staging, monitoring, surveillance, prediction or prognosis. The **clinical role** of a test explains its position relative to existing tests in the clinical pathway. A replacement test, for example, replaces an existing test. A triage test is used before an existing test; an add-on test is used after an existing test.

Besides diagnostic accuracy, several other outcomes and statistics may be relevant in the evaluation of medical tests. Medical tests can also be used to classify patients for purposes other than diagnosis, such as staging or prognosis. The STARD list was not explicitly developed for these other outcomes, statistics, and study types, although most STARD items would still apply.

DEVELOPMENT

This STARD list was released in 2015. The 30 items were identified by an international expert group of methodologists, researchers, and editors. The guiding principle in the development of STARD was to select items that, when reported, would help readers to judge the potential for bias in the study, to appraise the applicability of the study findings and the validity of conclusions and recommendations. The list represents an update of the first version, which was published in 2003.

More information can be found on http://www.equator-network.org/reporting-guidelines/stard.

CC BY SA
